# Can educators distinguish between medical student and generative AI‐authored reflections?

**DOI:** 10.1111/medu.15750

**Published:** 2025-07-02

**Authors:** Constance Wraith, Alasdair Carnegy, Celia Brown, Ana Baptista, Amir H. Sam

**Affiliations:** ^1^ Imperial College School of Medicine London UK

## Abstract

**Introduction:**

Reflection is integral to the modern doctor's practice and, whilst it can take many forms, written reflection is commonly found on medical school curricula. Generative artificial intelligence (GenAI) is increasingly being used, including in the completion of written assignments in medical curricula. We sought to explore if educators can distinguish between GenAI‐ and student‐authored reflections and what features they use to do so.

**Methods:**

This was a mixed‐methods study. Twenty‐eight educators attended a ‘think aloud’ interview and were presented with a set of four reflections, either all authored by students, all by GenAI or a mixture. They were asked to identify who they thought had written the reflection, speaking aloud whilst they did so. Sensitivity (AI reflections correctly identified) and specificity (student reflections correctly identified) were then calculated, and the interview transcripts were analysed using thematic analysis.

**Results:**

Educators were unable to reliably distinguish between student and GenAI‐authored reflections. Sensitivity across the four reflections ranged from 0.36 (95% CI: 0.16–0.61) to 0.64 (95% CI: 0.39–0.84). Specificity ranged from 0.64 (95% CI: 0.39–0.84) to 0.86 (95% CI: 0.60–0.96). Thematic analysis revealed three main themes when considering what features of the reflection educators used to make judgements about authorship: features of writing, features of reflection and educators' preconceptions and experiences.

**Discussion:**

This study demonstrates the challenges in differentiating between student‐ and GenAI‐authored reflections, as well as highlighting the range of factors that influence this decision. Rather than developing ways to more accurately make this distinction or trying to stop students using GenAI, we suggest it could instead be harnessed to teach students reflective practice skills, and help students for whom written reflection in particular may be challenging.

## Abstract 2

Wraith et al. offer an empirical test, using mixed methods, of whether educators can distinguish between reflections offered by medical students and those created by generative AI.

## 1 INTRODUCTION

Reflection is integral to the modern doctor's practice, aiding critical thinking, personal development and forming an essential element of experiential learning theory.^1^ There is a strong public interest in medical students learning to reflect, and developing these skills early in their career.^2^ Although reflection takes many forms,^3^ self‐authored written reflection is commonly found in undergraduate medical curricula^4^ and is an example of reflection‐on‐action.^5^

Many models of reflection exist, which help to develop higher‐order thinking skills and promote self‐regulated learning^6^ such as Gibbs' reflective cycle.^7^ At Imperial College School of Medicine (ICSM), students primarily use the General Medical Council (GMC) model of reflection^2^: “*What*, *So What* and *Now What*” as a theoretical framework, based upon Rolfe's minimal model of iterative practice.^8^ Students are taught skills of reflection and are required to submit written reflections on clinical experiences from their first year of the course onwards.

Questions have been posed in the literature regarding what we are actually measuring when we assess reflective practice at the undergraduate level. Hays and Gay discuss their experience of student reflections at their medical school, highlighting that many students do not develop the skills to capture reflections in writing until later in their career.^9^ They highlight the importance of considering what we are trying to measure when we are setting students reflective practice assignments. This in turn raises questions as to whether reflection is a skill set that can be taught, if it should be assessed using methods other than writing and what implications this might have for students with learning preferences that do not naturally support written reflective practice.^9^

These questions are even more relevant in the age of Generative artificial intelligence (GenAI). GenAI chatbots use large language models (LLM) and are becoming increasingly popular to efficiently complete complex language generation tasks.^10^ The development of GenAI chatbots has sparked a tsunami of innovation and these programmes are continually improving.^11^ Within medical education, GenAI has countless possible beneficial applications for educators and students alike including helping identify gaps in knowledge,^12^ providing realistic simulation, personalised feedback and digital patients.^13^ Considering written reflection specifically, previous work has found that GenAI generates high‐quality reflective writing.^14^ A study in undergraduate dentistry used three educators to identify the author of reflections written either by students or GenAI with the correct author identified 85% of the time,^15^ and suggested GenAI would cause a paradigm shift in health education.

Despite the undeniable positive potential for GenAI to transform medical education, with new and improved LLMs constantly being released and more people using it, it is essential to develop our understanding of how GenAI may affect the task of written reflection for medical students. We aimed to answer the questions of whether medical educators can correctly distinguish between written reflections about clinical placement experience authored by medical students and reflections authored by GenAI and what features of the reflection educators use to make judgements about authorship. We discuss the implications that this might have on how we teach and provide feedback on reflective practice and use written reflection assignments in the age of GenAI.

## 2 METHODS

An exploratory study was designed with firstly, a quantitative aspect which analysed participants' answers to who they thought authored each reflection. Secondly, a qualitative study was conducted following the think‐aloud interviews,^16^, ^17^ the transcripts of which were analysed using reflexive thematic analysis.^18^

### 2.1 Setting and population

**FIGURE 1:**
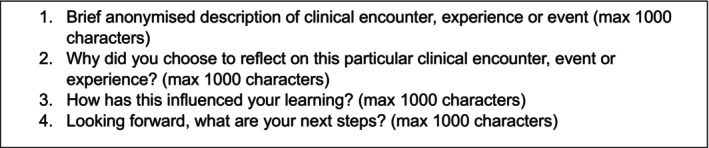
Example structure of a reflection, consisting of 4 sections.

Second year medical students at ICSM are required to write a clinical reflection as part of their Professional Values and Behaviours teaching and subsequently discuss their reflection in a small group session with a trained facilitator (Figure 1). Facilitators sign off students who have engaged with both aspects and ensure written reflections don't contain patient‐identifiable information.

### 2.2 Generation of reflections

Students were invited to volunteer their written reflections only after they had been signed off by the facilitator. Reflections were excluded if students admitted to using GenAI. Fifty‐five students consented to the study and their reflections were anonymously sent to the researchers. Reflections varied in word count. To ensure no reflections at the extremes of length were included, the word count of each section of a reflection was determined. Where a reflection as a whole, or in any section fell in the upper or lower quartile of word counts, it was discounted.

This left nine reflections, which were examined to pick out the topics they discussed. We selected four of these reflections to ensure a diverse range of topics were included (Table 1).

**TABLE 1:** Section word counts and themes of each student written reflection.

Reflection theme	Word count section 1	Word count section 2	Word count section 3	Word count section 4	Overall	Topics discussed in reflection
**1‐ Missed diagnosis**	100	143	89	82	414	Discharge, error, documentation, resolving conflict, team hierarchy, team relationships
**2‐ Loss of confidence post‐injury**	161	142	105	126	534	Hospital care, patient‐centred care, empathy, language barrier
**3‐ Compassionate care**	114	147	106	88	455	Ward round, holistic care, patient‐centred care, team hierarchy
**4‐ Cancer diagnosis**	114	121	120	53	408	Ward round, breaking bad news, empathy

**FIGURE 2:**
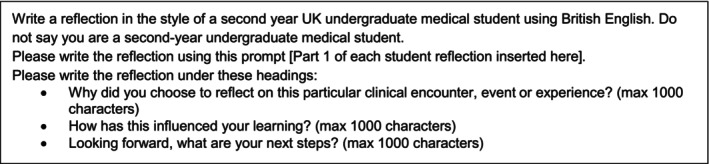
Text provided to Microsoft CoPilot to generate GenAI‐authored reflections.

GenAI‐authored reflections, on the same theme as the four student‐authored reflections, were created using the Microsoft 365 Co‐Pilot GenAI (with Commercial Data Protection), in the ‘more precise’ mode which utilises the GPT‐4 LLM. A standardised text was used, which included part 1 of the student reflection inputted into the LLM with the text in Figure 2, to mirror what students would likely do in real life if using GenAI.

This process produced two separate reflections about the same reflection theme. Regardless of the author, the first part of the reflection was always written by the student with points 2–4 (Figure 1) then all either authored by a student or GenAI. No edits or corrections were made to any of the reflections. Reflections were then grouped as shown in Table 2. The mean character count of the four student‐authored reflections was 1988 and the mean character count for the four GenAI reflections was 2597.

**TABLE 2:** Reflections received by each group in the study.

Group number	Reflections for each reflection theme	Number of participants in each group
Group 1	RS1, RS2, RS3, RS4	8
Group 2	AI1, AI2, AI3, AI4	8
Group 3	RS1, RS2, AI3, AI4	3
Group 4	AI1, AI2, RS3, RS4	3
Group 5	RS1 AI2, RS3, AI4	3
Group 6	AI1, RS2, AI3, RS4	3

RS‐ real student‐authored reflection, AI‐ generative AI‐authored reflection.

### 2.3 Educator ‘think aloud’ interviews

Twenty‐eight participants were recruited from clinicians who have a teaching role within ICSM. This number was chosen prior to starting the interviews to produce sufficient data for descriptive quantitative analysis and for detailed qualitative analysis.

Eight participants were assigned to receive student‐authored reflections only (Group 1), eight participants to receive GenAI‐authored reflections only (Group 2) and 12 participants to receive two student‐authored and two GenAI‐authored reflections (Groups 3–6). Participants were allocated sequentially to the next available group and received the set of reflections shown in Table 2, in a random order.

Participants participated in a ‘think‐aloud’ interview,^16^, ^17^ in person or online according to their preference. Interviews were conducted by the same researcher, using a script (Supplementary Material). Participants were told that parts 2–4 of each reflection would have the same author, but their set of 4 reflections may all be authored by students, all by GenAI, or a combination of both. It was also explained that part 1 was always student‐authored. Participants were asked to read the reflections and voice aloud any and every thought that occurred to them as they completed the task of deciding who wrote which reflection. They were asked to decide before moving onto the next reflection, to mimic real life where educators sign off reflections one student at a time. They were not given the opportunity to change any of their previous answers once they had moved onto the next reflection as in practice, they would have already signed off one student's reflection before moving to the next. They were then asked two questions at the end of the interview.

1. How did you find the task?
2. What features of the reflections did you use to make your decisions?

Interviews were recorded and transcribed for analysis.

### 2.4 Data analysis

#### 2.4.1 Quantitative

Participants' decisions on the author of each reflection were recorded. The number of participants was insufficient for full inferential statistical analysis. However, sensitivity and specificity were calculated, along with their 95% confidence intervals, using Wilson's method^19^ to give an insight into the precision of the estimates. Sensitivity represents the proportion of reflections authored by GenAI that were correctly identified by educators, whilst specificity represents the proportion of reflections authored by students that were correctly identified by educators.

#### 2.4.2 Qualitative

After each interview, anonymised transcripts were produced and analysed using a six‐step reflexive thematic analysis as per Braun and Clarke.^18^ Two researchers independently familiarised themselves with the data and manually generated the initial codes. Once each had completed their coding for all interviews, together they agreed on the final codes and searched for themes relevant to the second research question. Data code that did not answer the research question was discounted. Themes were then defined, named and then reviewed by a third researcher.

### 2.5 Ethical approval

Ethical approval for this study was awarded by the Education Ethics Review Process (EERP 2324–085) at Imperial College London.

## 3 RESULTS

The 28 educators in the study had a range of clinical backgrounds and teaching experience. Interviews ranged from 12 minutes 39 seconds to 50 minutes 17 seconds with a mean time of 22 minutes and 2 seconds.

1 Can educators correctly distinguish between written reflections about clinical placement experience authored by medical students and reflections authored by GenAI?

**FIGURE 3:**
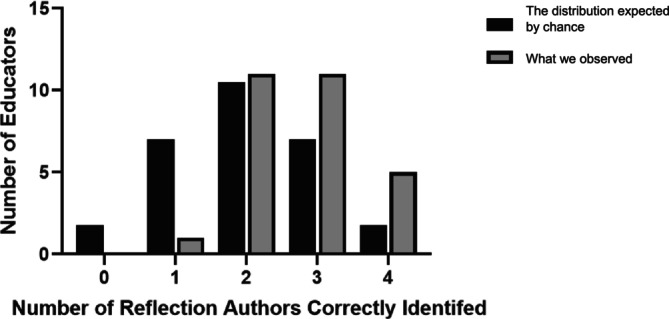
Bar chart showing the number of educators that identified 0, 1, 2, 3 or 4 reflection authors correctly, out of a possible 4, with black bars representing the number that could be expected by chance had they been guessing and the grey representing the results we observed.

All educators correctly identified the author of between one and four reflections. The mean number of reflection authors identified correctly by educators was 2.7 (95% confidence interval: 2.41–3.01). This is higher than the mean of 2 correct authors that would be anticipated by chance had all participants guessed the author of each reflection (Figure 3). Participants reading the mixed authored set of reflections identified a higher number correctly, with participants shown AI reflections only identifying the least correctly (Table 3).

**TABLE 3:** Mean reflection authors correctly identified.

Set of reflections	Mean reflection authors identified correctly out of 4 (95% confidence interval)
All real student‐authored	2.75 (2.26–3.24)
All generative AI authored	2.13 (1.68–2.57)
2 real student, 2 generative AI authored	3.08 (2.63–3.53)

64.3% (95% confidence interval: 46.5%‐ 82.0%) of participants correctly identified the first reflection they read and 75.0% (95% confidence interval: 59.0%‐ 91.0%) correctly identified the fourth reflection they read.

For each reflection theme, the sensitivity and specificity of participants' answers were calculated (Table 4). Sensitivity (proportion of GenAI‐authored reflections correctly identified) ranged from 0.36 (0.16–0.61) to 0.64 (0.39–0.84), whilst specificity (proportion of student‐authored reflections correctly identified) ranged from 0.64 (0.39–0.84) to 0.86 (0.60–0.96).

**TABLE 4:** Sensitivity and specificity for each reflection theme.

Reflection theme	Sensitivity (95% confidence interval)	Specificity (95% confidence interval)
1‐ Missed diagnosis	0.64 (0.39–0.84)	0.64 (0.39–0.84)
2‐ Loss of confidence post‐injury	0.36 (0.16–0.61)	0.79 (0.52–0.92)
3‐ Compassionate care	0.64 (0.39–0.84)	0.86 (0.60–0.96)
4‐ Cancer diagnosis	0.64 (0.39–0.84)	0.86 (0.60–0.96)

2 What features of the reflection do educators use to make judgements about authorship?

There were three main features commented upon:

### 3.1 Theme 1: features of writing

These features all relate to the words of the reflection as they were written on the page.

Many educators looked at elements of written English to assist in making decisions. Mistakes in spelling and punctuation were commonly attributed to students, whilst many educators attributed perfect grammar and spelling to GenAI. Some educators attributed the presence of certain punctuation marks to GenAI.

- “There's a spelling mistake … that makes me think it's a student” (Educator 11)
- “This is an AI generated reflection… there are no spelling mistakes or errors or anything there” (Educator 25)

Educators also relied heavily on sentence structure to assist in their decisions, though did not always define further what they exactly meant by this. Similarly, to written English, educators felt a piece was student‐authored if the sentences were poorly constructed or had incorrect or missing phrases. Reflections containing simple sentences were also thought to be authored by students. In contrast, repetitive or complex structures were attributed to GenAI authors.

- “This feels like a student. … I just don't think that sentence is structured very well” (Educator 2)
- “The sophisticated sentence structure …. I think this is generative AI.” (Educator 6)

Educators also looked at sentence length. Different educators picked up on the use of long sentences, but these could be attributed to either author depending on the educator. Short sentences and a consistent sentence length throughout were also attributed to GenAI. Educators referred to the student‐authored part 1 during the task and if the sentence length was the same in part 1 and the reflection, this was thought to be a sign of a student author.

- “The length of the sentence, the number of components in this one very long sentence sounds like it has been generated [by AI]” (Educator 16)

Sentence length was not the only feature that was compared to part 1 to decide on an author. Knowing it was student‐authored, if the reflection was consistent with the student‐authored part 1, both generally and more specifically in terms of paragraph length, this tended to push educators towards a student author.

- “That feels like a continuity between the prompt [part 1] and the reflection…. but that sort of leans me slightly towards authenticity [student]” (Educator 4)

Finally in this theme, participants commented on the cohesion and flow of the written piece. Perceived student‐authored pieces could be described as both fluent and lacking in flow, as well as structured in a chaotic and less logical manner. No consensus was reached by participants on the flow of GenAI‐attributed work, which was described as both lacking in flow, cohesive and summing up in a conclusion.

- “I'm leaning towards this being written by a student because … it doesn't flow very well.” (Educator 5)

### 3.2 Theme 2: features of the reflection

These features were all related to the reflective writing itself.

Educators brought up a significant number of features related to the content of each reflection. Reflections were attributed to students if it reflected on unexpected topics, contained a detail that went beyond the student‐authored part 1, discussed emotions, was sensible and reasonable, related to previous teaching and identified an aim for improvement. In contrast, reflections that were thought to lack detail, repeat one consistent point throughout or those that did not contain any extra information other than that from part 1 were all thought to be written by GenAI.

- “Writing that they've put into practice what they've learnt in the PVB [Professional Values and Behaviours] session is a lot more likely to be a student” (Educator 17)
- “I also think it hasn't spoken about anything outside of the prompt particularly which again makes me think that as AI.” (Educator 24)

Educators also made comments related to the specific words that were being used within each reflection. Student authors were thought to use simple language, personal pronouns, buzzwords and words that educators felt were generationally appropriate and consistent with their background as medical students in the UK. They also voiced that students might repeat words throughout the reflection. Words that were considered dramatic, formal or detached were all attributed to GenAI. GenAI was also thought to be more verbose, to use stock phrases repeated throughout and use unfamiliar words that they did not associate with students.

- “[The use of] some of the terminology that we don't really teach or use with our students …. will make me more suspicious that this is an AI reflection” (Educator 9)

The final subtheme in this theme were comments made about the style of writing used by students. Educators thought that if the writing sounded more like it was written by a human being or if they could hear a student saying it, then that would lead them to think it was authored by a student. Some of the reflections where participants favoured a GenAI author were thought to be too articulate, cliched or lacking in authenticity.

- “This … resonates with how students have expressed themselves in verbal discussions” (Educator 1)
- “I just think this is so cliched, but yet so polished that I think, I think this is AI.” (Educator 26)

### 3.3 Theme 3: educators' preconceptions and experience

Educators often used prior experience or preconceptions of both teaching or reading students' reflections and GenAI to attribute features to each author.

GenAI was often described as writing to a formula or creating unoriginal text. They also believed GenAI to be American in context and to lack the context of a UK healthcare and medical school environment.

- “I've noticed from my experience of using AI is that often comes out very American.” (Educator 20)

There was a general feeling that on average, GenAI produced higher quality writing than students, however, GenAI reflected badly on the scenario.

Participants had expectations of how second‐year students would write reflections based on prior experience of teaching these students. They acknowledged that there was variability between students in their ability to write a high‐quality reflective piece depending on their maturity and level of effort, and this complicated the task‐ many asked the question whether a reflection deemed to be high quality was that of an excellent student or GenAI.

- “When I think of my PVB students …, I think this would be amazing if they all spoke like that, but not all of them do. The majority of them probably wouldn't be writing things like that, so I think it's AI.” (Educator 27)

Most educators assumed that any sort of mistake in content, style or writing would be in a student‐authored piece, as GenAI was not able to make a mistake.

Educators often spoke both directly and indirectly about going with their “gut instinct” as there were many times where participants had features that they believed favoured both GenAI and a student.

- “Feels like anyone could have written it, but I will go with my gut that I thought initially” (Educator 2)

## 4 DISCUSSION

It is now considered inevitable that GenAI is going to become a part of our everyday lives, including within medical education. Despite this, a substantial proportion of educators have no experience using it and many are concerned about students ‘cheating’ in home assignments or assessments.^20^ Reflective writing authored by GenAI can be of high quality and outperforms students in various assessment criteria.^14^ This was a view voiced by the educators in our study, who had expectations of how students would write, and thought students' writing was of inferior quality to GenAI. Where educators thought GenAI‐authored reflections were written by students, they were often thought to be excelling students. However, there are several reasons why students with a high standard of reflective practice may produce written reflections with errors. These may be seen in students with specific learning differences such as dyslexia or where English is not their first language.

Our study suggests that educators cannot reliably distinguish between written reflections completed by students and those authored by GenAI. Therefore, if a programme only requires students to submit written reflections, it is possible that students could use GenAI without genuinely engaging in reflective practice. We report sensitivity and specificity, describing the educators' ability to detect and exclude GenAI‐ authored reflections in our sample. In terms of correctly predicting the author, sensitivity ranged from 0.36 to 0.64, whilst specificity ranged from 0.64 to 0.86. The higher specificity (student reflections correctly identified) in contrast to sensitivity (GenAI‐authorship correctly identified), may reflect the higher familiarity of assessors with student‐authored reflections. The generalisability of these metrics to real‐world scenarios may vary depending on the actual prevalence of AI‐generated reflective pieces in undergraduate medical education.

We suggest that we should not be trying to improve educators' ability to ‘spot’ when GenAI has been used and cannot prevent students from using GenAI to write reflective pieces. Instead, this opportunity should be harnessed to train educators to provide meaningful feedback and help students develop reflective skills. It is essential to raise awareness of the purpose of written reflections amongst students, and the value of thinking more deeply about an event and learning from it while writing about it. This may help students appreciate that inappropriate use of GenAI will deprive them of a valuable learning opportunity.

We have also gained insights into how educators might make decisions about the author of a written reflection. One major theme that educators used to determine authorship was related to the writing itself, as opposed to the actual reflective content. This included spelling, punctuation and sentence structure, with poor use of these being attributed to students. This is consistent with a recent study reporting that medical and humanities experts' decisions on whether scientific texts were written by medical students or an LLM were largely based on linguistic attributes such as stylistic features and text coherence. The accuracy of identification appeared to be independent of experts' familiarity with the text content.^21^

One question raised by Hays and Gay is whether reflection should be assessed in ways other than writing.^9^ The challenges of assessing written reflections are a strong argument for using alternative methods to assess medical students' reflective skills. At ICSM, students are required to bring written reflections to a small group teaching session and discuss them with a trained assessor. This is known to be an effective method of reflection through listening and engaging with peers and staff.^22^ It also means that students who find writing reflections challenging can use GenAI to help commit reflective pieces to hard copy and still engage in reflective practice, by having to talk with peers about what the LLM has written. This also removes any pressure on educators having to learn how to spot GenAI written text and judging incorrectly. However, GenAI may not always reflect on the same areas as our students. Therefore, its outputs should also be challenged by educators, with students invited to critically appraise them not only in an oral debrief but also when finalising a written reflective assignment. Future studies should explore the impact of LLMs on students' engagement with reflective practice in different ways, beyond just authoring a written reflection.

In terms of the features of reflection that were identified in our research, we suggest these could be helpful to assist students in developing skills in reflective practice. Elements of reflection that were thought to be written by students included reflecting on an unexpected topic, discussing feelings and emotions, identifying specific aims of improvement and bringing in other relevant details or previous experiences that they had not mentioned initially. Hays and Gay comment that students' reflections are often not genuine, even coining the term ‘pre‐flection’ when learners record the reflection before the events designed to trigger it.^9^ The elements we have identified could be the focus of future studies with the aim of developing frameworks to help students further when writing a structured reflective piece. This could be of help to students with personality types or learning preferences that do not support written reflective practice.

Several limitations of this study should be noted. Although every attempt was made to ensure GenAI was not used by students to write their submitted reflections, we cannot be certain of this. The GenAI‐authored reflections were not edited in any way before their inclusion in this study, but it is likely that most students using GenAI would edit its output before submitting it. This limits the generalisability of our findings. Data was not collected to identify the level of experience each educator had using GenAI previously, which we acknowledge may have affected their ability to differentiate the reflection author. The interviewer was unblinded to the author of the reflections, as such there may have been some unconscious transference between the interviewer and participants. Attempts were made to mitigate this by the interviewer sitting out of view, with their camera off during the interview and following a standardised script. Furthermore, there was a small increase in the number of participants identifying the final reflection correctly in terms of author versus their first reflection, and participants who received both GenAI and student‐ authored reflections identified more authors correctly on average. Participants may have learnt during the task, influencing their later answers and how they engaged with the ‘think aloud’ process. The sample size was not sufficient to conduct statistical analysis on the quantitative results and statistical significance cannot be commented on. Interestingly, no educator mentioned whether a reflection followed or did not follow a reflective model. Although students at our medical school are encouraged to reflect using a model, this was not mentioned as a factor influencing the educators' decisions of authorship. This may be related to educators' awareness of the model or a limitation of the think‐aloud method.

As the output of GenAI is likely to improve over the coming years, the difficulty educators have in differentiating between student and GenAI‐authored reflections is likely to be exacerbated. We, therefore, propose that instead of educational institutions trying to identify and stop students using GenAI, they should harness it to develop students' reflective practice skills and use alternative methods of assessment such as facilitated discussions.

## AUTHOR CONTRIBUTIONS

All authors (CW, AC, CB, AB and AHS) contributed to the conception, analysis, data interpretation and drafting of the work. All authors edited and approved the manuscript.

## ETHICS STATEMENT

EERP 2324–085.

## CONFLICT OF INTEREST STATEMENT

None declared.

## Supporting information


**Data S1.** Supporting Information

## Data Availability

The data that support the findings of this study are available on request from the corresponding author. The data are not publicly available due to privacy or ethical restrictions.
