# Supplementary material for: Can educators distinguish between medical student and generative AI‐authored reflections?
Source: Med Educ. 2025 Jul 2;59(11):1214–21. doi: 10.1111/medu.15750 (PMC12513547; doi:10.1111/medu.15750)
Supplement: Supplementary file 1 — Data S1. Supporting Information [file MEDU-59-1214-s001.docx]

**Supplementary Material: ‘Think Aloud’ Interview Script**

Adapted from Leighton JP. Using think-aloud interviews and cognitive labs in educational research: understanding qualitative research. New York: Oxford University Press; 2017

Thank you for attending the session.

- Please can I confirm you have read the participant information sheet and are happy to participate in the study today?
- Do you have any initial questions?

As described in the information sheet, we are conducting a study focussed on understanding how educators distinguish between AI and student written reflections. Today I will ask you to read through four reflections on the clinical experiences of Phase 1b medical students. Each reflection will begin with a prompt [part 1] which has always been written by a student, followed by 3 paragraphs. These three paragraphs will either have been written by a student, or generative AI, but not both.

I will show you 4 reflections in total which I will provide to you one at a time. These 4 reflections may all have been written by students, all by AI or a mixture of the two. Once you have read each one, I will ask you to tell me who you think wrote that reflection. Once we have moved onto the next reflection you will not be able to go back and change your previous answers. Once you have finished reading and given me your answers, I will ask you two questions about the task.

The reflections will be displayed on your screen one at a time. Please let me know when you would like to move onto the next reflection and if you need me to change the font size, font colour or background colour to help with your reading.

I am interested in learning about the thoughts you have as you answer so will ask you to think aloud as you answer. By ‘think aloud’ I would like you to tell me everything you think as you work through each reflection.

When I say tell me everything, I really mean every thought you have from the moment you start to read the first reflection to the end when you give me your thoughts on who wrote the final reflection.

Please do not worry about planning how to say things or clarifying your thoughts. What I really want is to **hear your thoughts constantly** as you read the reflections – uninterrupted and unedited. Sometimes you may need time to think quietly through something which is absolutely fine but please tell me what you thought through as soon as possible after you are finished.

I realise it can feel awkward to think aloud but try to imagine [you are alone in the room/ I am not on the call]. If you become silent for too long, **I will say “keep talking” to remind you to think aloud**. The purpose of the study is to learn about the thoughts you—and other people—have as you read the reflections, rather than evaluate these thoughts.

The whole call will be recorded and transcribed so we can analyse what thoughts you have during the task. We will delete the recording once transcription has taken place. Please feel free to turn your camera off if you wish to, as we are only analysing the audio. I will turn off my camera during the interview, unless you request, I keep it on. Should you wish to, you are able to withdraw from the interview at any point, just let me know and we will stop. Do you have any questions? Are you happy to go ahead?

I will now show you a practice task to get you used to reading and thinking out loud.

Both a human and generative AI were asked to describe a fox in one sentence. Please read both and tell me who you think wrote which, thinking aloud as you do so:

- A fox is a small to medium-sized, omnivorous mammal with a bushy tail, pointed ears, and a reputation for being cunning and adaptable in various habitats.
- A fox is a mammal that lives in urban environments, is mainly nocturnal, has distinctive orange colouring and is intelligent.

**Interviewer should not give the answer.**

We will now move onto the task. Can you see the first reflection? Are you happy with the font size, colour and background colour? This is reflection number 1. The prompt was written by a student and the next three paragraphs wither by a student, or generative AI.

**Once a participant has given their answer:** Are you finished with this reflection; would you like to move on?

**If yes:** This is reflection number [2-4]. The prompt was written by a student and the next three paragraphs wither by a student, or generative AI.

**The interviewer should intervene only with keep talking after periods of silence longer than 10 seconds. The interviewer will not ask participants to elaborate.**

**At the end of interview ask the participants:**

- How did you find the task?
- What features of the reflections did you use to make your decisions?

Thank you, that is the end of the interview.
